# Weather, Not Climate, Defines Distributions of Vagile Bird Species

**DOI:** 10.1371/journal.pone.0013569

**Published:** 2010-10-22

**Authors:** April E. Reside, Jeremy J. VanDerWal, Alex S. Kutt, Genevieve C. Perkins

**Affiliations:** 1 Climate Adaptation Flagship and Ecosystem Sciences, Commonwealth Scientific and Industrial Research Organisation, Townsville, Queensland, Australia; 2 Centre for Tropical Biodiversity and Climate Change, School of Marine and Tropical Biology, James Cook University, Townsville, Queensland, Australia; University of Zurich, Switzerland

## Abstract

**Background:**

Accurate predictions of species distributions are essential for climate change impact assessments. However the standard practice of using long-term climate averages to train species distribution models might mute important temporal patterns of species distribution. The benefit of using temporally explicit weather and distribution data has not been assessed. We hypothesized that short-term weather associated with the time a species was recorded should be superior to long-term climate measures for predicting distributions of mobile species.

**Methodology:**

We tested our hypothesis by generating distribution models for 157 bird species found in Australian tropical savannas (ATS) using modelling algorithm Maxent. The variable weather of the ATS supports a bird assemblage with variable movement patterns and a high incidence of nomadism. We developed “weather” models by relating climatic variables (mean temperature, rainfall, rainfall seasonality and temperature seasonality) from the three month, six month and one year period preceding each bird record over a 58 year period (1950–2008). These weather models were compared against models built using long-term (30 year) averages of the same climatic variables.

**Conclusions:**

Weather models consistently achieved higher model scores than climate models, particularly for wide-ranging, nomadic and desert species. Climate models predicted larger range areas for species, whereas weather models quantified fluctuations in habitat suitability across months, seasons and years. Models based on long-term climate averages over-estimate availability of suitable habitat and species' climatic tolerances, masking species potential vulnerability to climate change. Our results demonstrate that dynamic approaches to distribution modelling, such as incorporating organism-appropriate temporal scales, improves understanding of species distributions.

## Introduction

Impacts of climate change on species are frequently predicted by projecting species distribution models (SDM) onto future climate change scenarios. Meaningful predictions of species' distributions require SDM to closely reflect a species' environmental limits and requirements; that they reflect the species' ecological niche [1]. Traditionally, SDM predict the geographic distribution of suitable climatic space for a species by relating species occurrence records to long-term average climate variables. Such models are generally a good representation of a species' broad range [2], as species are closely connected to climatic conditions through exchanges of energy and mass [3], [4]. The standard approach to SDM incorporates climate variables such as mean annual temperature and annual precipitation averaged over periods of c. 30 years [5]. SDM generated for climate change predictions commonly use a baseline period of 1961–1991 [6], [7], [8]. The use of long-term climate averages in SDM has an ecological basis when modelling sessile or sedentary organisms such as plants [9], [10]. However, the application of SDM to more mobile species requires investigation of the appropriateness of long-term climate averages as a one-size-fits-all approach. A small number of studies have accounted for the dynamic nature of species distributions by including climatic variables corresponding to migratory species' arrival and breeding times [11], and other relevant breeding times [12], based on a priori knowledge of species movements. However, for mobile species with less predictable movement patterns, a new approach is needed. The temporal scales important to a mobile individual's location are likely to be much shorter than a 30 year average [13], [14]; therefore short-term weather may be more appropriate.

Models tailored to incorporate organism-specific temporal scales are important when modelling species which respond to fluctuations in resource availability following short-term weather events. Weather (defined as the conditions over a short period, for this study it represents a period of 12 months or less) and climate (long-term average, >20 years) both play a large role in the movement of mobile species in search of food and breeding opportunities [15], [16], [17]. However, weather might play a proportionally greater role in movement patterns in regions characterized by high variability in conditions, particularly where rainfall is both variable and limiting [13]. Variable rainfall prevents species from relying on regular seasonal migrations to find suitable conditions, instead favouring flexible resource-tracking behaviours [18]. Resource tracking is a common trait among birds found within the tropical savannas of northern Australia [19], [20], a region characterised by highly seasonal rainfall, and variable inter-annual weather patterns [21], [22]. The life history of many species is linked with the pulses of nectar, fruit and insect abundance following rainfall events [14], [17], [19], [20].

In addition to climatic variability, Australian land birds are not subject to severe winter conditions like those experienced by their northern hemisphere counterparts [18]. The relatively benign winters and stochastic weather patterns within Australia shift the balance in a hypothetical cost-benefit trade off between staying versus relocating from year to year [13], resulting in complex local and continental scale migratory patterns [23]. The flexible nature of movement patterns of Australian birds is reflected in the array of different movement classifications found in the literature [23], [24], [25], [26], [27]. Up to 19 distinct movement patterns have been identified, and many species show variation within populations and across years [23]. Four main categories are consistently used, despite the blurred boundaries between the groupings [18]: *migration* is the predictable seasonal movement from a breeding ground to a wintering ground and return within the year; *nomadism* is wandering to wherever conditions are suitable, with yearly variations in routes and distances taken; *sedentary* species remain in the same locality throughout the year; and *partial migration* occurs when some individuals within a species migrate and others are sedentary.

We hypothesise that SDM built using short-term weather variables (weather models) will outperform models built using long-term climate averages (climate models), and this improvement will be strongest for species responding to stochastic weather events. Species more responsive to stochastic weather events are generally nomadic, and in particular those with large distributions covering many biogeographic regions, moving in search of suitable conditions. In particular, the arid zone faces substantial boom-bust cycles [28], so species associated with arid zones are likely to be particularly responsive to weather patterns. Distributions of 157 bird species were modelled using means and seasonality of temperature and precipitation representing either weather (three, six and twelve month values immediately preceding date of a bird sighting) or climate (30 year average representing 1961–1990). We tested whether a species' range size, biogeographic affiliation or movement strategy influenced the relative importance of weather vs. climate variables in defining a species distribution. Range sizes were defined as small (see methods for details), medium or large, and biogeographic affiliations were temperate, tropical and arid [29]. A further affiliation, “ubiquitous”, was included to account for species encompassing two or more zones. Species were classified into a movement category: sedentary, nomadic and partially migratory; and a category combining species which are both sedentary and nomadic. Very few species could be classified as true migrants within our study area (see methods) so were not included in this study. Model performance (defined as the models' discriminancy and consistency [30]) was evaluated by the area under the receiver operator curve (AUC): an AUC score of 1 is a perfect fit of the data, 0.5 is no better than random [5], [31].

## Results

**Figure 1:**
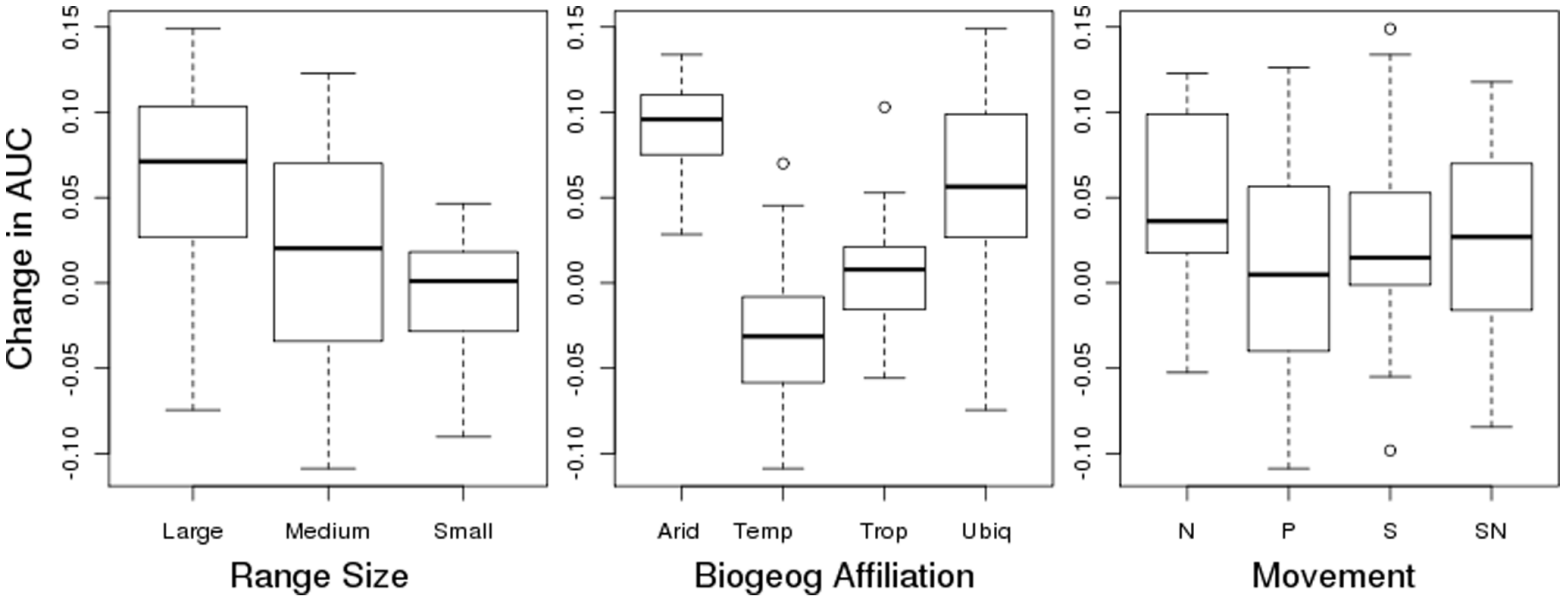
The pairwise differences in AUC values for all species between weather and climate models. Boxplots (mean ±25th and 75^th^ percentiles) show species are grouped by range size biogeographic region and movement patterns (see methods for description of classes). In most cases the change is positive – showing an improvement of AUC for weather models when compared with the climate models.

Our results show that short-term weather provides a significant improvement in modelling bird distributions. Overall, weather models outperformed climate models, with a mean improvement in the model test statistic AUC of 0.026 (Wilcoxon signed rank test *p*<0.00001). Model fit differed between climate and weather models depending on range size, biogeographic region and movement (Figure 1). AUC increased more for wide ranging species than for either small or medium-ranging species (Kruskal-Wallis ANOVA *p*<0.0001). When comparing AUC values for species across their biogeographic zones, weather models outperformed climate models to a greater extent for arid species, followed by ubiquitous and tropical species (Kruskal-Wallis ANOVA, *p*<0.0001). Weather models outperformed climate for species in three of the four biogeographic zones (Figure 1). Temperate species showed a mean negative AUC difference; therefore climate models on average slightly outperformed the weather models for these species. The change in AUC across movement categories identified that nomadic species improved the most, followed by sedentary and then partially migratory species (Kruskal-Wallis ANOVA, *p* = 0.0001). Predictably, the group of species which are both sedentary and nomadic sat in between the purely nomadic and purely sedentary species.

**Figure 2:**
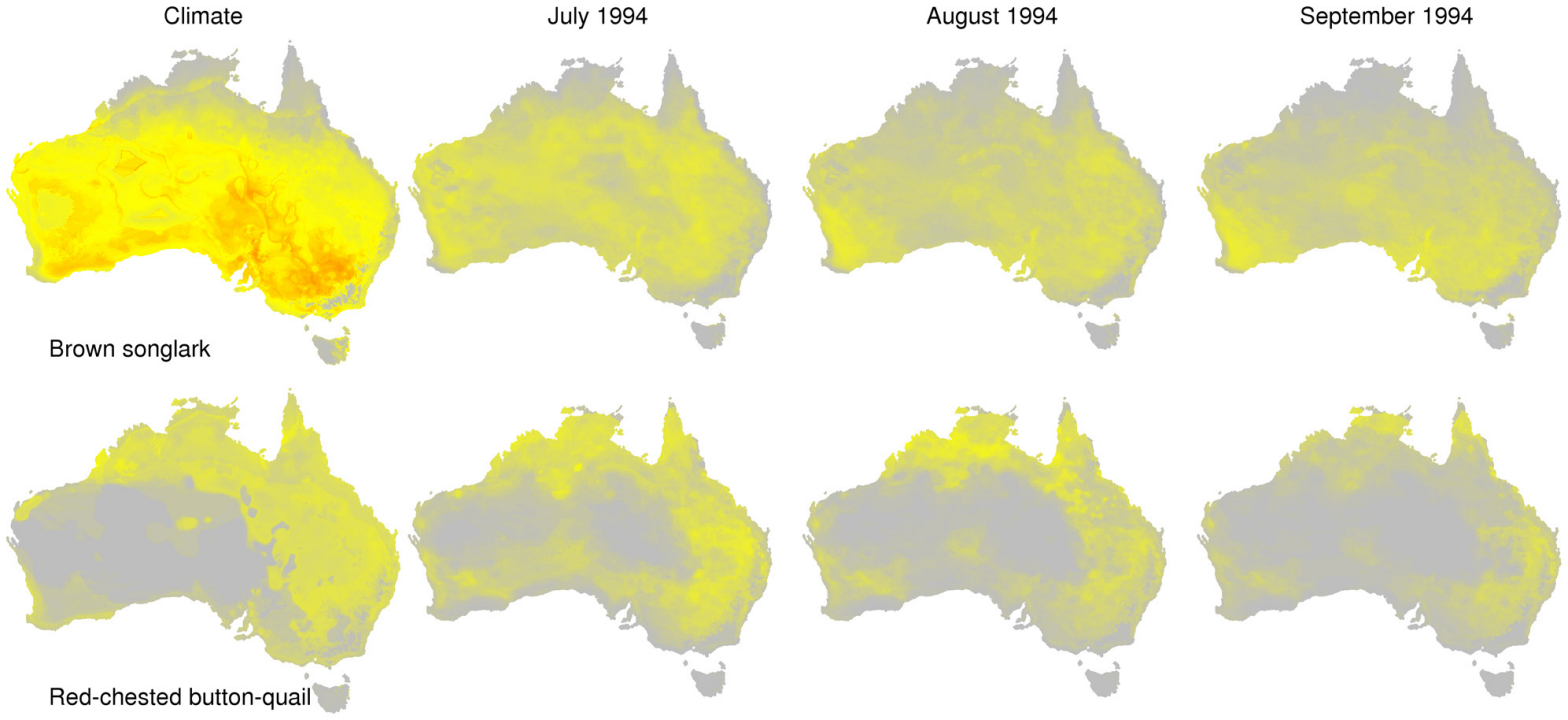
The predicted range for two species determined by the climate model and the weather model. The ranges are shown for brown songlark (*Cincloramphus cruralis*) and the red-chested button-quail (*Turnix pyrrhothorax*). For illustration purposes, the weather model was projected onto 3 consecutive months to illustrate the changes in the distribution of suitable area depending on the weather conditions for a particular month. The probability distribution is shown for each particular month, with grey unsuitable, and increasing suitability shown from yellow to orange (most suitable).

**Figure 3:**
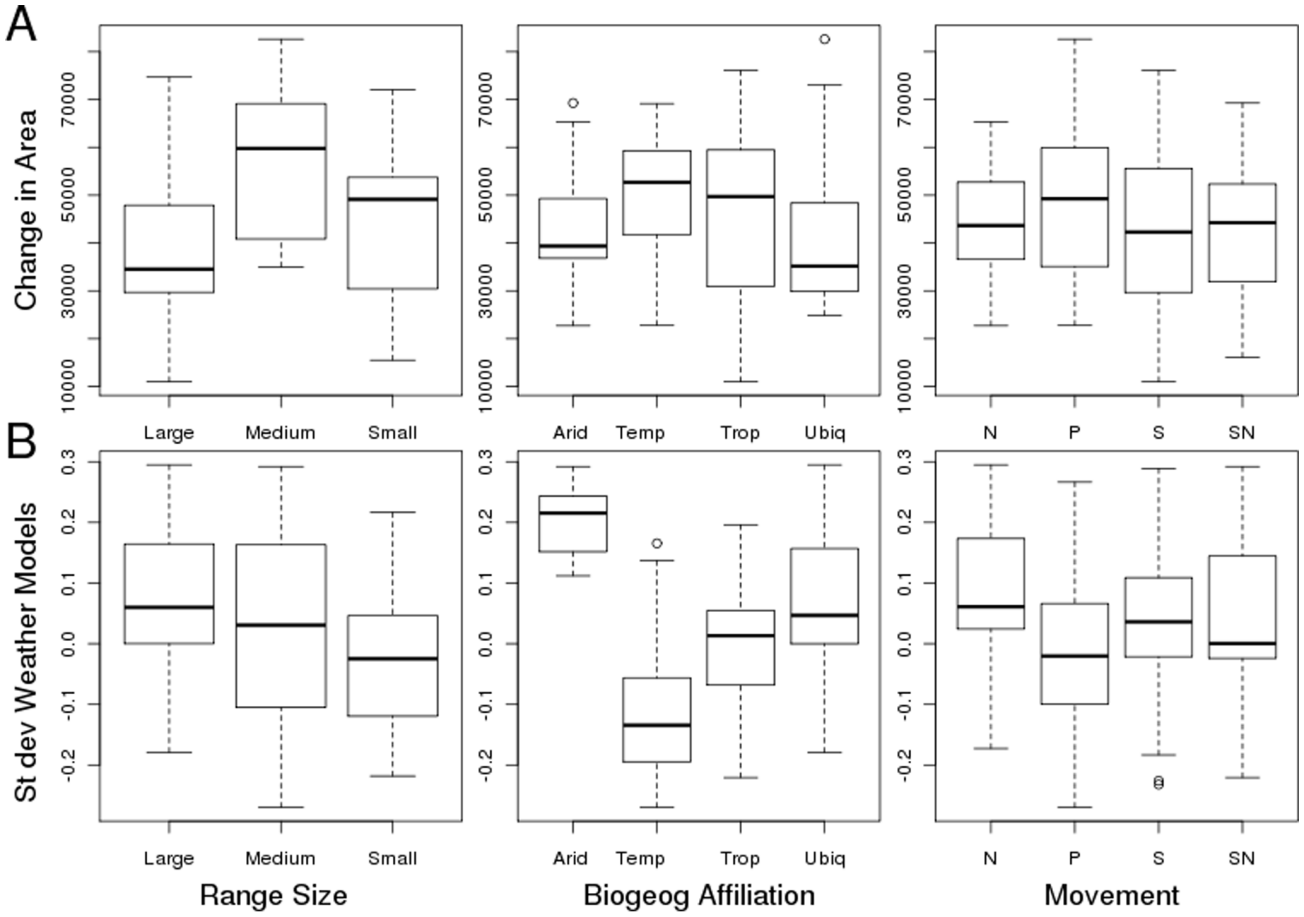
Area predictions for climate and weather models across range sizes biogeographic zones, and movement categories. (*A*) The difference in predicted area (mean ±25th and 75^th^ percentiles). (*B*) The standard deviation of area for weather models projected onto each month for the period 1950 – 2008, compared across range size biogeographic zone, and movement categories.

Weather models give a more refined understanding of the extent and location of suitable conditions both seasonally and inter-annually, when compared with the distributions generated using long-term climate averages. An example of monthly fluctuations is shown in Figure 2 for two birds, the brown songlark (*Cincloramphus cruralis*) and the red-chested button-quail (*Turnix pyrrhothorax*). The weather models demonstrate substantial fluctuations in the distribution of suitable habitat available across months, compared to predictions based on long-term climate averages. The difference in area of suitable habitat for each species as predicted by weather and climate models changes significantly when comparing range sizes (Kruskal-Wallis ANOVA, *p* = 0.0001), biogeographic affiliations (*p* = 0.0001) and movement categories (*p* = 0.0002) (Figure 3*A*).

The extent of fluctuation (standard deviation) of suitable conditions when projected onto each month from 1950 – 2008 also varies significantly across these groups (Figure 3B). Species with small ranges show little difference between climate and weather area predictions and little fluctuation in area across months. Species with medium ranges show the largest fluctuations, and geographically widespread species show the biggest difference in predicted area yet the least fluctuations across months (Kruskal-Wallis ANOVA, *p*<0.0001). When species were grouped by biogeographic zones, climate and weather models differed the most for arid and ubiquitous species, but the least fluctuations month to month for weather model predictions (*p* = 0.0076). There was no difference in range fluctuations for species in different movement categories (*p* = 0.4612) It is therefore the wide-ranging, ubiquitous and arid species that have the most marked difference between climate and weather area predictions, the weather models predicting less area, and the least fluctuation across months. By contrast, suitable habitat area predicted for narrow-ranging, temperate and tropical species is quite similar for both weather and climate models.

**Figure 4:**
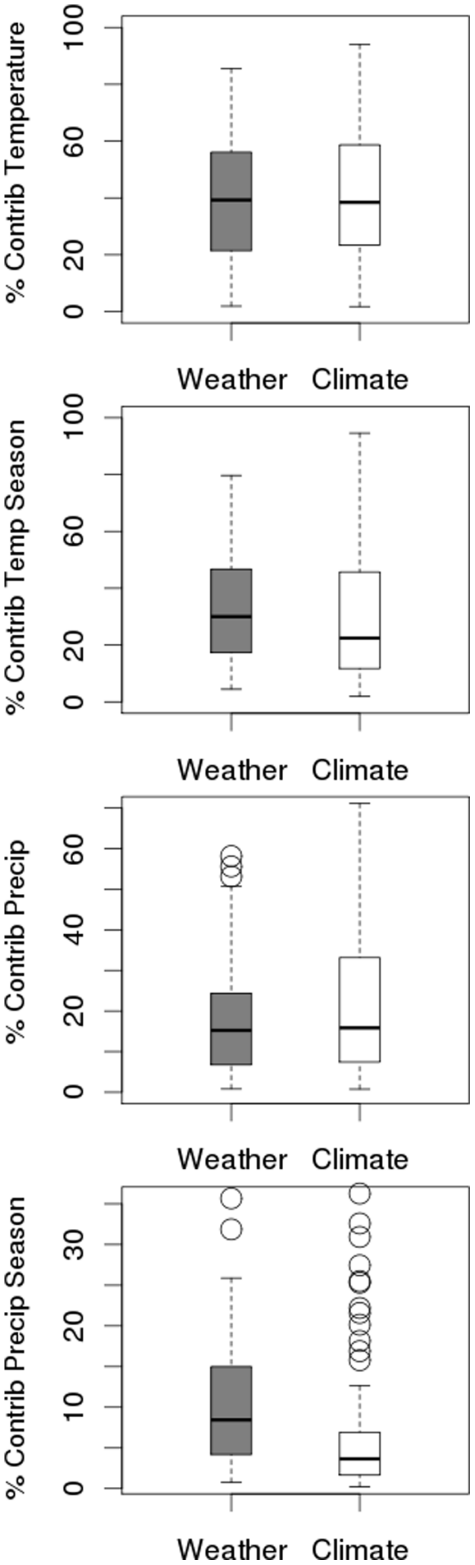
The contribution of different variables to the weather and climate models. mean temperature, temperature seasonality, precipitation and precipitation seasonality(mean ±25th and 75^th^ percentiles). Bars representing weather are shown in grey, while bars representing climate are white.

Altering the temporal scale of the model variables from 30 year to six month and one year periods changes the relative contributions of the variables. Precipitation contributed significantly more to climate models (Wilcoxon matched pairs test, *p* = 0.003), and precipitation seasonality contributed significantly more to weather models (*p*<0.001) (Figure 4). Temperature was on average the most influential variable across for both climate and weather models, followed by temperature seasonality, precipitation and then precipitation seasonality. We examined the differences in variable contribution to models depending on a species' life history, and how this changed with temporal scale. All variables contributed differently for species across biogeographic affiliations to the *p*≤0.01 level (Kruskal-Wallis ANOVA; Figure S1). For the other life history characteristics, results were varied. The contribution of temperature differed significantly according to range size (climate models: *p* = 0.061; weather models: *p* = 0.019), and the contribution of precipitation differed according to movement (climate models: *p* = 0.01; weather models: *p* = 0.024).

Examination of variable contribution to models reveals substantial differences for species within each biogeographic zone (Table 1). Mean temperature contributed most to climate models for temperate, arid, and ubiquitous species, whereas temperature seasonality was the most influential for the weather models for species within these zones. The reverse was true for tropical species, as climate models were most influenced by temperature seasonality, and mean temperature was the highest contributor for weather models. The difference in variable contribution for models of species across biogeographic zone was significant for 12 out of 16 cases. As “seasonality” refers to the coefficient of variation for the time period, it is evident that variability in temperature and rainfall are more influential upon bird distributions over short time periods.

**Table 1:** The differences in the variable contributions (mean temperature, temperature seasonality, mean precipitation, precipitation seasonality) to climate and weather models for species across biogeographic zones.

Zone	Variable	Climate Mean	Weather Mean	t-value	df	*p*
Tropical	Mean Temp	28.12	**52.25**	−6.73	106	**0.000**
	Temp Season	**48.01**	24.14	6.14	106	**0.000**
	Precipitation	**20.46**	14.58	1.83	106	0.070
	Precip Season	3.42	**9.03**	−4.80	106	**0.000**
						
Temperate	Mean Temp	**52.23**	35.00	3.81	66	**0.000**
	Temp Season	11.42	**30.35**	−6.30	66	**0.000**
	Precipitation	**29.24**	20.81	2.22	66	**0.030**
	Precip Season	7.11	**13.84**	−4.06	66	**0.000**
						
Arid	Mean Temp	**40.02**	23.47	2.86	30	**0.008**
	Temp Season	39.08	**43.76**	−0.70	30	0.487
	Precipitation	15.57	**25.81**	−2.15	30	**0.040**
	Precip Season	5.33	**6.97**	−1.18	30	0.248
						
Ubiquitous	Mean Temp	**47.81**	36.58	2.38	104	**0.019**
	Temp Season	23.00	**37.85**	−4.17	104	**0.000**
	Precipitation	**21.36**	15.53	2.09	104	**0.039**
	Precip Season	7.84	**10.05**	−1.38	104	0.169

The bold values in the climate and weather mean columns are the higher value; bold values in the *p* column indicate a significant difference at the 0.05 level.

## Discussion

Successful species distribution models (SDM) require appropriate temporal correspondence between species records and environmental variables [31], [32]. This correspondence is muted by using long-term climate averages for vagile species, reducing the ability to produce accurate models [9], [33], [34]. Here we demonstrate a technique that uses an organism-relevant temporal scale to model species. The approach more precisely reflects the scope and variability of species' environmental requirements and habitat suitability. We find that weather models largely outperform climate models, and this improvement is most apparent for wide-ranging, nomadic and desert species, and species that traverse multiple biogeographic zones. These species are likely to be the most responsive to weather events and corresponding resource fluctuations [35]. Interestingly, weather models also outperformed climate models for sedentary species. Sedentary species may undertake local movements in response to weather events [14], and their distribution might be limited by climatic extremes [10], two factors that are better accounted for with weather models than climate. Extreme conditions, for example a period of extreme high temperatures coupled with rainfall deficits, may limit species directly due to their own biological threshold [10], [36] or indirectly by limiting food or other habitat resources [37].

Weather models did not outperform climate models or result in larger predicted areas for species with smaller ranges or for temperate species. This suggests that these species' ranges fluctuate less in the short-term; in other words, these species are not tracking resources on this temporal scale and are therefore better explained by long-term averages. Temperate and more restricted species are likely to be adapted to local conditions [16], possibly by diet switching rather than relocating when resource availability changes [14], [38]. Evidence for diet flexibility has been shown for small-range species in both tropical [39] and temperate Australia [40].

Climate models predicted a greater amount of suitable space than weather models for most species. The larger distributions predicted by climate models result in an over-estimate of the availability of suitable habitat. In contrast, the weather models refine the suitability criteria of an area by incorporating the temporal component to produce smaller predicted areas. Weather models identify the shifting environmental suitability within the species' broader climatic range, and that suitability shifts across time. Climate models have the assumption that a location is always suitable for a species if the species was recorded there. However this assumption is invalid for species that undertake large movements to find suitable conditions because of vast temporal fluctuation in conditions [41]. Climate models mask the highly fragmented distributions of key refugial habitats during a resource nadir, leading to an inflated understanding of a species' resistance to extinction. This over-estimation of species' ranges leads to inaccurate assessments of species conservation status, vulnerability to climate change and the degree of protection existing conservation reserves provide.

Robust predictions of the impacts of climate change on species require SDM that account for temporal fluctuations in habitat suitability. Climate change predictions for northern Australia include increasing climatic variability with greater frequency and severity of extreme events [42], [43], and increase in drought conditions due to increase in temperature, decrease in rainfall and increased evapotranspiration [44]. Tropical savanna bird species have adapted to highly variable conditions through plastic migratory and nomadic movement behaviour [18]. However, flexible movement behaviour relies on patches of suitable conditions being within reasonable proximity, because movement has inherent risks and food supplies must be found before energy stores drop critically low [45], [46]. Increased climatic variability in combination with increased drought conditions could result in areas of suitable conditions becoming further apart in space, or staying unsuitable for longer periods of time. Vagile species may therefore need to move more frequently to find new suitable conditions. While predictions have been dire for species with narrow niche requirements, such as those relying on montane microclimates [47], [48], wide-ranging species have attracted much less concern. However, species undertaking large-scale movements for specific niche requirements may be vulnerable to increased weather variability. This may result in suitable conditions becoming more energetically expensive to find [45]. Highly dispersive land birds, such as specialist nectarivores the regent honeyeater (*Xanthomyza phrygia*) and the swift parrot (*Lathamus discolour*) have shown declines due to anthropogenic changes in the landscape [14], [41], [49]. Increased variability may lead to other vagile species, such as those in tropical savannas, showing similar declines. Changing patterns of climatic variation are likely to be the crucial element of species persistence, especially in highly variable areas. Therefore, modelling techniques that do not incorporate the short-term weather fluctuations are likely to underestimate climate change implications.

Examining the relative contributions of the different variables to the model can help tease out what is most influential to species distributions. Temperature is known to have a great influence on where a species can occur due to thermal constraints on energetics [3]. It is interesting to note that temperature seasonality, the coefficient of variation of temperature across a given time period, was also highly influential to species' ranges. This highlights that it is both the mean temperature and the variations in temperature that are highly influential to species' ranges. As expected, the contribution of each variable differs across biogeographic affiliations, as the regions themselves differ climatically.

Including temporal variations in habitat suitability of mobile species is essential for understanding a species' actual conservation status, and how well the species is protected by conservation reserves. Mobile species present both monitoring and conservation challenges [50], as suitable habitat may need to be retained in geographically disjointed locations [33], [50], [51], [52]. Maintenance of suitable habitat for mobile species will require an extensive reserve network in conjunction with conservation-compatible non-reserve land management to accommodate species that track suitable conditions [50], [53]. Conservation planning needs to incorporate dynamic processes, particularly shifting species distributions [54], [55]. This is evident for northern Australia [50], however it is also true for the conservation of vagile species in other parts of the world including the northern hemisphere [56]. Our weather modelling technique is a tool for greater understanding of species range dynamics and therefore vital to conservation planning for mobile species.

The need to incorporate specific climatic conditions relevant to species in order to accurately model distributions is increasingly being recognised [11]. Both spatial [57], [58] and temporal heterogeneity [11], [12] are being incorporated into models, but accounting for interannual climatic variability is still largely lacking. Accounting for this variability is likely to be important to a range of taxa. Studies have shown that species across taxonomic groups: from plants, insects, fish, amphibians, reptiles, mammals and birds; have all shown shifts in distributions in the direction expected as a response to climate change, albeit at different temporal scales [59]. This suggests that species' ranges are responding to fluctuations in climate; therefore accounting for this in SDM is important. Birds are an extreme example of mobility compared to other terrestrial species due to their lower costs of transport [3], however many marine species (particularly pelagic species, and including marine mammals) are highly dispersive [60] and correlating their occurrences to their physical environment at short-term intervals is likely to greatly increase model performance and understanding of their distributions. Mobile mammals such as ungulates undertake movements in response to rainfall fluctuations [61], and other volant and dispersive species, such as bats and some invertebrates, are likely to be responsive to weather variation [50], [62]. While the benefits of incorporating short-term weather in SDM may be intuitive for highly dispersive species, we have shown that the weather models also outperformed the climate models for sedentary bird species, suggesting that our weather modelling technique could be beneficial for species more affected by short-term weather fluctuations and variability than long-term averages. This may be particularly true of species with short generation times.

Our results show that understanding how species respond to weather conditions over short- and medium-term temporal scales is essential for quantifying species climatic limits. It is also important for understanding species' responses to rapid climate change and understanding their conservation status. Modellers should consider temporal scales appropriate to their organism when generating SDM and making climate change predictions.

## Materials and Methods

Over four million occurrence records of 157 Australian tropical savanna bird species were collated across the period 1950 to 2008 from the Birds Australia Atlas [63], [64], the Queensland Governmental atlas WildNet [65] and CSIRO (protocol as in [66]). The data were spread across the time period (Figure S2). Species' range sizes were defined as either small (less than two million square kilometres; *n* = 62), medium (between two and four million square kilometres; *n* = 53), and large (greater than four million square kilometres; *n* = 42). Species range sizes were those reported in the New Atlas of Australian birds [64] and these categories appeared to adequately represent the spread of species' ranges. Species were categorised by movement life history (nomadic (*n* = 25), sedentary (*n* = 54), partially migratory (*n* = 59), and species that were both nomadic and sedentary (*n* = 19)) according to the literature [15], [67], [68], [69], [70], [71], [72]. The study focused on bird species using the savannas, therefore excluded waterbirds and rainforest species. Species with inadequate data coverage were excluded from the analyses. Due to the blurred boundaries between movement categories [25], [38], [73], we only used species that could be reliably characterized as nomadic, sedentary, both nomadic and sedentary, or partially migratory in the movement comparisons; very few species within this assemblage could be classified as true migrants and therefore were not the focus of this study. All species in the study occur within Australian tropical savannas, however most species also occur over large areas outside this region. Species were therefore also categorized by which biogeographic affiliation best described their overall range, based on those defined by [29] (original names in parentheses): tropical (Torresian), for distributions across northern Australia (*n* = 54); temperate (Bassian), for those down the eastern and southern coastal woodlands and forested areas (*n* = 34); arid (Eyrean), for those predominantly in arid inland Australia (*n* = 16); and ubiquitous for species that encompassed two or more of the above categories (*n* = 53).

Daily precipitation and temperature minima and maxima from 1950 until 2008 at a 0.05° grid scale were accessed from the Australian Water Availability Project (AWAP) [74], [75]. From this, we calculated annual mean temperature, temperature seasonality, annual precipitation and precipitation seasonality over the baseline period of 1961 – 1990 as our climate data. Temperature and precipitation seasonalities were the coefficient of variation over the given time period. Our weather data were created by calculating the above variables for three, six, nine, twelve months; and three, six, nine years previous to each month that a bird was recorded within the period 1950 to 2008. We reduced the number of variables to minimize the chance of over-fitting the model. We removed variables three, six and nine years as they were all highly correlated with one year (see Table S1 A). We also removed nine months for all variables and three months for mean temperature and precipitation due to the correlations between the months (see Table S1 B). Although the remaining variables (six months and one year for all variables, and three months for temperature and rainfall seasonalities) still had some high correlations, the SDM algorithm can handle such correlation [76].

Species distribution models were run using the presence-only modelling program Maxent [31]. Maxent uses species presence records to statistically relate species occurrence to environmental variables on the principle of maximum entropy. The climate data contained unique combinations of latitude and longitude for each species and the corresponding values for the four climate variables. The weather data files consisted of each unique combination of month, year, latitude and longitude of each bird sighting, and the corresponding weather or climate variables for each relevant time period (three, six and twelve months, depending on the variable). All default settings were used except for background point allocation. Background points (pseudo-absences) can be selected in a number of ways; here we used a target group background [77]. By using the locations and dates of all bird records (‘target group’) as our background points, it is assumed that any sampling bias (spatially or temporally) in our occurrence records for a single species can also be observed in our background points; in effect cancelling out the effect of any spatial or temporal sampling bias in the modelling exercise. The models were projected onto spatial surfaces consisting of the model variables across Australia for each calendar month between 1950 and 2008. AUC is potentially influenced by the number of model variables, as increasing the number of variables can lead to an over-fitted model. We investigated whether the higher AUC of our weather models was due to the greater number of variables than in our climate models by running our weather models for each species with only four weather variables. The proportional increase in AUC of the four-variable weather model to the climate model, compared with the AUC increase of the weather model to the climate model, was 0.83 (±0.11 SE). Therefore on average, 83% of the AUC improvement was due to the weather models having a better fit of the data, and 17% of the increase in AUC was due to the increased number of variables.

Threshold values based on balancing training omission rate, predicted area and logistic threshold value were incorporated to convert the Maxent default probability distribution to a binary presence/absence [78]. This provided realistic predictions of species distributions [79], [80]. The threshold was read in from the Maxent Results output file, so that every pixel in the ascii output above the threshold was counted as “presence”, and every pixel below the threshold was scored as “absence”. The mean and standard deviation of suitable area (measured as the number of 5° cells, which is roughly equivalent to 58,000 km^2^) were then calculated for each species. We used Wilcoxon signed rank tests to compare model performance of weather vs. climate models, as this method is widely used for comparing AUCs in similar studies [6], [10], [12]. Wilcoxon signed rank tests were also used for comparing the difference in variable contribution between weather and climate models. When comparing AUCs, area, or variable contributions of weather and climate models against a grouping variable, we used the Kruskal-Wallis ANOVA. We used 0.05 as our alpha-level significance value. Analyses were conducted using R version 2.9.0 (www.r-project.org) and Statistica version 8.

## Supporting Information

Figure S1 The contribution of the different variables to the weather and climate models. The contribution of the variables depending on range size, biogeographic affiliation and movement classification (mean ±25th and 75th percentiles). Bars representing weather are shown in grey, while bars representing climate are white.
(2.68 MB TIF)

Figure S2 Histogram of bird records across the time period of 1950 to recent.
(0.69 MB TIF)

Table S1 (0.01 MB DOCX)
