# Supplementary figures and images for: Weather, Not Climate, Defines Distributions of Vagile Bird Species
Source: PLoS One. 2010 Oct 22;5(10):e13569. doi: 10.1371/journal.pone.0013569 (PMC2962630; doi:10.1371/journal.pone.0013569)

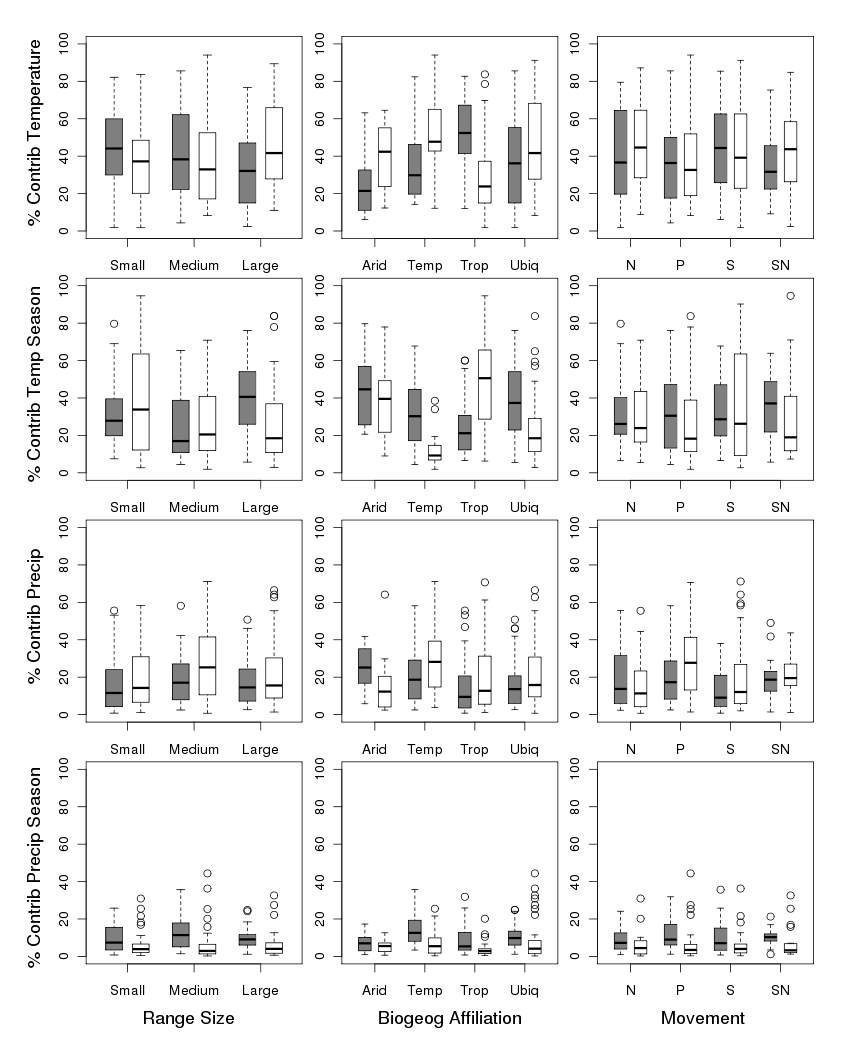

Supplement: Figure S1 — The contribution of the different variables to the weather and climate models. The contribution of the variables depending on range size, biogeographic affiliation and movement classification (mean ±25th and 75th percentiles). Bars representing weather are shown in grey, while bars representing climate are white. (2.68 MB TIF) [file pone.0013569.s001.tif]

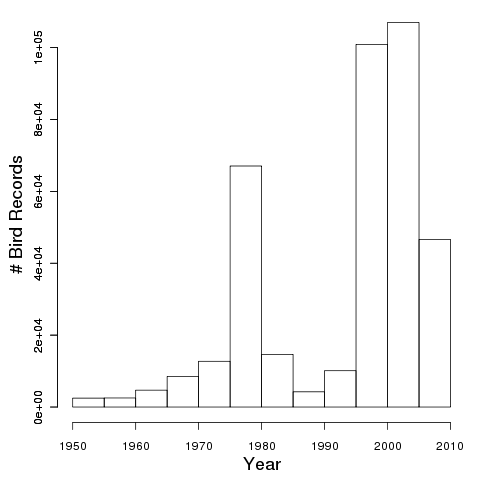

Supplement: Figure S2 — Histogram of bird records across the time period of 1950 to recent. (0.69 MB TIF) [file pone.0013569.s002.tif]
