# Supplementary material for: Weather, Not Climate, Defines Distributions of Vagile Bird Species
Source: PLoS One. 2010 Oct 22;5(10):e13569. doi: 10.1371/journal.pone.0013569 (PMC2962630; doi:10.1371/journal.pone.0013569)
Supplement: Table S1 — (0.01 MB DOCX) [file pone.0013569.s003.docx]

**Table S1.** The correlation matrices for four climate variables over different time periods. A) The correlations between each of the four variables between the four time periods in years (one, three, six and nine years). B) The correlations between the four different time periods in months (one, three, six and nine months).

| A) | Mean Temp | Temp Seasonality | Precipitation | Precip Seasonality |
| --- | --- | --- | --- | --- |
|  |  |  |  |  |
|  | 1 yr | 1 yr | 1 yr | 1 yr |
| 3 yr | 0.997 | 0.967 | 0.995 | 0.873 |
| 6 yr | 0.997 | 0.963 | 0.992 | 0.819 |
| 9 yr | 0.996 | 0.951 | 0.981 | 0.724 |
|  |  |  |  |  |
| B) | Mean Temp | Temp Seasonality | Precipitation | Precip Seasonality |
|  |  |  |  |  |
|  | 1 m | 1 m | 1 m | 1 m |
| 3 m | 0.986 |  | 0.93 |  |
| 6 m | 0.937 | 0.828 | 0.866 | 0.675 |
| 9 m | 0.901 | 0.643 | 0.839 | 0.506 |
